# The Effect of Serum Calcium Levels on Uremic Encephalopathy in Patients with Acute Kidney Injury in the Emergency Department

**DOI:** 10.3390/medicina55050204

**Published:** 2019-05-23

**Authors:** Canan Akman, Dilek Ülker Çakır, Serkan Bakırdöğen, Serdal Balcı

**Affiliations:** 1 Emergency Medicine Department, Çanakkale Onsekiz Mart University Faculty of Medicine, 17020 Çanakkale, Turkey; drcananakman@gmail.com; 2 Biochemistry Department, Çanakkale Onsekiz Mart University Faculty of Medicine, 17020 Çanakkale, Turkey; ducakir@gmail.com or ducakir@yahoo.com; 3 Internal Medicine Department, Çanakkale Onsekiz Mart University Faculty of Medicine, 17020 Çanakkale, Turkey; sbdogen@comu.edu.tr or serkanbakirdogen@hotmail.com; 4 Şırnak State Hospital, Emergency Service, 73000 Şırnak, Turkey

**Keywords:** acute kidney injury, serum calcium, uremic encephalopathy

## Abstract

*Background and objectives*: Uremic encephalopathy is the most important complication of renal failure and urgent dialysis treatment is required. Parathormone (PTH) contributes to the etiopathogenesis of uremic encephalopathy. PTH is a hormone that acts in the calcium balance in the organism. The aim of our study was to investigate the effect of serum adjusted and ionized calcium on the development of uremic encephalopathy in patients with acute renal injury (acute kidney injury network (AKIN) stage 3). *Materials and Methods*: Our study was supported by Canakkale Onsekiz Mart University Scientific Research Projects Unit (ID:1278). Three groups were formed for the study. The first group was acute renal failure AKIN stage 3 (N: 23), the second group was AKIN stage 3, and the patients who had emergency hemodialysis (N: 17) and the third group (N: 9) had AKIN stage 3 hemodialysis due to uremic encephalopathy. In these patient groups, 25-hydroxy vitamin D, PTH, calcium, albumin, urea, creatinine, and blood-gas-ionized calcium were observed in their serum during the first application. Calcium, albumin, urea, creatinine, and ionized calcium in blood gas were also examined in serum at 24th and 72th hours. Data were analyzed using SPSS version 19.0. Kruskal–Wallis test and Mann–Whitney U test were applied for the variables that did not comply with normal distribution. *p* < 0.005 was accepted statistically. *Results*: A statistically significant difference was found between the measurement creatinine values at the 24th and 72th hours of admission in AKIN stage 3 patients who applied to the emergency department (*p* = 0.008). A statistically significant difference was found in the measured calcium values (*p* = 0.013). A statistically significant difference was found in the measured ionized calcium values (*p* = 0.035). *Conclusions*: In our study, the effect of ionized calcium level on uremic encephalopathy in serum creatinine, calcium, and blood gas in patients presenting with acute renal injury, AKIN stage 3, was significant, but studies with new and large groups are needed.

## 1. Introduction

Acute kidney injury (AKI) may be induced by several causes. In spite of its high incidence, it is a treatable clinical syndrome. It exhibits a large spectrum from minor changes in the renal functions to the renal replacement therapy. 

AKI appears by the malfunction of kidneys in hours and days, accompanied with the reduced rate of glomerular filtration. As a result of the malfunction of kidneys, the metabolic products cannot be evacuated, which increase their serum level, and finally the uremia develops [1]. Emergence of uremia affects all the systems, and cases distortion of the calcium and phosphor metabolism, which is accompanied with the secondary hyperthyroid, whereupon some distortions in bone metabolism might develop [2]. Secondary hyperparathyroidism causes an increase in the cerebral cortex calcium content. It has been demonstrated in the uremic patients with secondary hyperparathyroidism patients that electroencephalography (EEG) changes are corrected upon repression of the serum parathyroid hormone (PTH) by medical or surgical treatment. Uremic encephalopathy has a complex pathophysiology, and many accumulated toxins contribute to the renal insufficiency. PTH takes place among the hormones which contribute to the uremic encephalopathy. As a result of the increased level of cytosolic calcium synaptosomes in the rats with chronic renal insufficiency, it is common to observe an increase in the total calcium content of the cerebral cortex. Increased cytological calcium in the rats with chronic renal insufficiency causes a decrease in the sodium-potassium adenosine triphosphatase (Na-K ATPase) and Ca ATPase activities, whereupon the synaptosomes inadequately respond to the depolarization resulting in distortion of the neurotransmitter metabolism [3,4,5].

Though AKI usually demonstrates a good moderate portrait, it may have a fatal risk depending on the severity of the underlying causes of AKI, and other accompanied diseases. For such reasons, an appropriate protective treatment and, when necessary, an appropriate and efficient renal replacement therapy must be planned for patients of AKI to prevent any potential complications of the uremia that may develop in this patient group [6].

In this context, this study aimed at detecting if the serum calcium level has an effect on the uremic encephalopathy in the patient group diagnosed of AKI.

## 2. Materials and Methods

AKI is characterized with a sudden distortion (within 48 h) in the renal functions, and an increase by >0.3 mg/dL in the serum creatinine level, and an increase of ≥50% (1.5 folds of basal) or decrease in the urine secretion (<0.5 mL/kg/s for longer than 6 h). In acute kidney injury network (AKIN) stage 3, an increase up to >300% in creatinine level (>3 folds of basal) or a serum creatinine level of ≥4 mg/dL—an acute increase for at least 0.5 mg/dL—with the urine secretion being <0.3 mg/kg/s for 24 h, or anuric for 12 h.

In this study supported by Scientific Project (BAP) (ID: 1278) of Canakkale Onsekiz Mart University, the patients were divided into three groups by AKIN stage 3 criteria. The first group of patients (Group 1) had AKIN stage 3 criterion with no dialysis treatment, and the second group (Group 2) underwent dialysis treatment with AKIN stage 3 criterion for any cause other than uremic encephalopathy, while the third group (Group 3) comprised of the patients who underwent dialysis for uremic encephalopathy according to AKIN stage 3 criteria. The study covered patients 18–80 years old. Any patients with the last-term renal insufficiency, with dialysis history, younger than 18 years and older than 80 years, were excluded from the study. The uremic encephalopathy was diagnosed upon a neurologic examination by the physician who evaluated the first patient in the emergency room. It was the nephrologist who decided for the studied patients to undergo dialysis or not.

All the patients admitted to the emergency room for the acute kidney injury in AKIN stage 3 table were checked up for serum calcium, albumin, urea, creatinine, 25-hydroxy vitamin D and PTH, and ionized calcium in blood gas at the time of admittance. Out of the patients hospitalized in the nephrology unit after the initial medical treatment in the emergency room, serum calcium, albumin, urea, creatinine, and blood gas calcium values were reviewed in 24 and 72 h.

In this study, the methods used to measure serum blood values include: Kinetic calorimetric test, based on Jaffe method, for creatinine; sandwich tests principle for PTH and electroluminescence bonding test for 25-hydroxy vitamin D; kinetic test with urease and glutamate (photometric) for urea; and colorimetric test and photometric measurement for albumin. Calcium ions, react with 5-nitro-5′metil-BAPTA to form a complex. The complex reacts with ethylenediamine tetraacetic acid (EDTA). The difference in absorbance is measured photometrically. All blood serum tests were performed in Roche/Hitachi Cobas C systems. Blood gas analyzers were used to measure ionized calcium. ABL 725 Radiometer Copenhagen device was used for assessment. ISE (ion-selective electrodes) were used for measurement.

The data were analyzed by the SPSS Package Software Version 19.0. In presentation of the descriptive data, the number, percentage, average, standard deviation, and minimum and maximum limits were used. Chi-square test was used in comparison of the categorical data. The quantitative data were compared by Kruskal–Wallis test and Mann–Whitney U test. It was adopted as *p* < 0.05 for the statistical significance. The ethical approval number for this study is ID: 1278.

## 3. Results

The study involved 49 subjects, including males by 42.9% (n = 21) and females by 57.1% (n = 28). The average age of patients was 71.4 ± 12.5 years with the median age being 73.0 (minimum: 25.0 years, maximum: 93.0 years).

Group 1, Group 2, and Group 3 comprised of male patients by 47.8% (n = 11), 52.9% (n = 9), and 11.1% (n = 1), respectively, as there was no significant difference among the groups in terms of gender distribution (*p* = 0.098).

The average age was 71.1 ± 15.0 years, 67.3 ± 8.3 years, and 80.1 ± 7.3 years with a significant statistical difference existing among the groups (*p* = 0.012). The average age of the Group 3 was higher than that of Group 2, and this difference is statistically significant according to Mann–Whitney U test with Bonferroni correction (*p* = 0.001).

A statistically significant difference was determined among the groups in terms of the average creatinine levels at the time of first admission in the emergency room (*p* = 0.008). The average creatinine level of Group 1 is lower than that of Group 2, and this difference is statistically significant according to Mann–Whitney U test with Bonferroni correction (*p* = 0.002). No statistically significant difference was found among the groups in terms of the average urea at the time of first admission in the emergency room (*p* > 0.05). A statistically significant difference was found among the groups in terms of the average albumin at the time of first admission in the emergency room (*p* = 0.045). In paired comparisons according to Mann–Whitney U test with Bonferroni correction, no significant difference was found among the groups in terms of the average albumin at the time of first admission in the emergency room (*p* > 0.05).

A statistically significant difference was found among the groups in terms of the average calcium at the time of first admission in the emergency room (*p* = 0.013). The average calcium in Group 1 was higher than that of Group 3, and it is statistically significant according to Mann–Whitney U test with Bonferroni correction (*p* = 0.007). The statistical difference was significant among the groups in terms of the ionized calcium values at the time of first admission in the emergency room (*p* = 0.035). The average ionized calcium in Group 1 was higher than that of Group 2, and it is statistically significant according to Mann–Whitney U test with Bonferroni correction (*p* = 0.012).

No significant difference was found among the groups in terms of the average PTH at the time of first admission in the emergency room (*p* = 0.270). There was no statistically significant difference among the groups in terms of average 25-hydroxy vitamin D at the time of first admission in the emergency room (*p* = 0.266) (Table 1).

There was a significant difference among the groups in terms of 24 h average creatinine (*p* = 0.001). The 24 h average creatinine of Group 1 was lower than that of Group 2, and it is statistically significant according to Mann–Whitney U test with Bonferroni correction (*p* = 0.0001). There was no significant statistical difference among the groups in terms of 24 h average urea (*p* = 0.354). There was a significant statistical difference among the groups in terms of 24 h average albumin (*p* = 0.045). The 24 h average albumin in Group 1 was higher than that of Group 3, and this difference is statistically significant according to Mann–Whitney U test with Bonferroni correction (*p* = 0.015). There was a statistically significant difference among the groups in terms of 24 h average calcium (*p* = 0.002). The 24 h calcium average in was is higher than that of Group 3, and this difference is statistically significant according to Mann–Whitney U test with Bonferroni correction (*p* = 0.001). The statistical difference was significant among the groups in terms of the average ionized calcium (*p* = 0.0001). The 24 h average ionized calcium in Group 1 was higher than that of Group 2 and Group 3, and it is statistically significant according to Mann–Whitney U test with Bonferroni correction (*p* = 0.0001 and *p* = 0.0001, respectively) (Table 2).

There was a significant difference among the groups in terms of 72 h average creatinine (*p* = 0.008). The 24 h average creatinine of Group 1 was lower than that of Group 3, and it is statistically significant according to Mann–Whitney U test with Bonferroni correction (*p* = 0.003). There was no significant statistical difference among the groups in terms of 72 h average urea (*p* = 0.626). There was a significant statistical difference among the groups in terms of 72 h average albumin (*p* = 0.005). The 72 h average albumin in Group 1 was higher than that of Group 3, and this difference is statistically significant according to Mann–Whitney U test with Bonferroni correction (*p* = 0.0003). There was a statistically significant difference among the groups in terms of 72 h average calcium (*p* = 0.001). The 72 h calcium average in Group 1 was higher than that of Group 3, and this difference is statistically significant according to Mann–Whitney U test with Bonferroni correction (*p* = 0.001). The 72 h calcium average in Group 3 was higher than the average of Group 1 and Group 2, and this difference is statistically significant according to Mann–Whitney U test with Bonferroni correction (*p* = 0.0001 and *p* = 0.009, respectively). The difference among the groups was statistically significant in terms of 72 h average ionized calcium (*p* = 0.0001). The 72 h average ionized calcium of Group 1 was higher than the average of Groups 2 and 3, and this difference is statistically significant according to Mann–Whitney U test with Bonferroni correction (*p* = 0.0001 and *p* = 0.001, respectively) (Table 3).

## 4. Discussion

AKI has a high rate of incidence in the community. The disease may, by nature, cause electrolyte disorders and some complications for the uremia. Such factors determine the hospitalization and treatment of the patients [7]. The patients’ average age in this study is high (>65), and it has been demonstrated by the other studies that the age is the most important factor of the AKI. There are many studies in the literature, revealing the high incidence of AKI among the patient older than 65 years [8].

In the patient groups diagnosed with chronic renal insufficiency, who has not yet undergone dialysis, a number of neurological symptoms were observed from light confusion to delirium and coma. Neural system dysfunction is an important medical problem. In addition to its role in the calcium and phosphor balance, PTH with increased serum levels in the patients of chronic renal insufficiency may also induce the uremic neurotoxin. The studies revealed that the cerebral calcium levels raise with the PTH effect in renal insufficiency.

The studies on synaptosomes have also demonstrated that PTH may affect the cerebral calcium movement [4,9]. In our study, serum PTH, and 25-hydroxy vitamin D hormone levels were found normal at the first admission in emergency room of AKIN stage 3 patients. However, in 24 and 72 h after admission, it was detected that both the level of serum total calcium and the ionized calcium levels in blood gas reduced. In our study, when the AKIN stage 3 patients were first admitted in the emergency room, it was found that both the serum total calcium and the ionized calcium levels in blood gas reduced more in Group 3 compared to the other groups. Furthermore, such low levels went on to be monitored even in the 24th and 72nd hours of admission, and maintained their statistical significance. At the time of the first admission, the Group 3 patients were observed to have higher creatinine levels and lower serum albumin levels compared to the other groups, while no significant statistical difference was found in the serum urea levels among the groups. In this context, this study reveal that the low levels of both the serum total calcium and the ionized calcium levels in blood gas might be useful in foreseeing if the uremic encephalopathy would develop in the patients with advanced level (AKIN stage 3) AKI patients.

The study had a number of limitations, however. No electroencephalography (EEG) could be performed in the patients who were admitted in emergency room with AKIN stage 3 and uremic encephalopathy, and underwent dialysis. In addition, no test was performed to measure calcium levels in the urine.

## 5. Conclusions

In the patients with AKI, who were admitted in emergency room for AKIN stage 3, the serum total calcium and low levels of ionized calcium in blood gas were important in foreseeing the development of uremic encephalopathy. The authors are of the opinion that there is a need of such prospective studies as to cover a higher number of patients.

## Figures and Tables

**Table 1:** Comparison of biochemical parameters of the groups at the time of first admission in the emergency room according to acute kidney injury network (AKIN) stage 3.

	Group 1 (n = 23)		Group 2 (n = 17)		Group 3 (n = 9)		
Biochemical Markers (Serum)	Mean ± SD	Median (min–max)	Mean ± SD	Median (min–max)	Mean ± SD	Median (min–max)	*P*
**Creatinine (mg/dL)**	4.4 ± 1.7	4.0 (2.6–7.8)	7.0 ± 3.1	6.2 (2.7–14.0)	5.3 ± 2.1	4.3 (3.2–9.6)	**0.008**
**Urea (mg/dL)**	181.2 ± 101.7	148.0 (74.0–532.0)	205.1 ± 116.2	183.0 (71.0–486.0)	182.1 ± 47.7	156.0 (126.0–273.0)	0.649
**Albumin (g/dL)**	3.8 ± 0.6	3.9 (2.5–5.1)	3.4 ± 0.8	3.4 (1.8–5.0)	3.2 ± 0.6	3.1 (2.5–4.0)	**0.045**
**Calcium (mg/dL)**	9.0 ± 0.7	9.2 (7.4–10.0)	8.5 ± 1.1	8.5 (5.7–10.5)	7.6 ± 1.8	8.5 (3.6–9.2)	**0.013**
**Ionized calcium (mmol/L)**	1.2 ± 0.1	1.2 (0.9–1.3)	1.1 ± 0.1	1.1 (0.8–1.5)	1.0 ± 0.2	1.1 (0.6–1.3)	**0.035**
**PTH (ng/mL)**	148.9 ± 81.8	134.0 (27.3–355.0)	186.1 ± 107.5	212.0 (14.2–368.0)	257.4 ± 230.9	224.0 (35.6–797.0)	0.270
**25-OH Vit D (ng/mL)**	16.3 ± 11.5	12.5 (3.5–38.8)	15.6 ± 8.1	16.4 (3.0–28.0)	10.9 ± 10.7	6.6 (3.0–30.7)	0.266

Mean: Average, SD: Standard deviation. PTH: Parathyroid hormone. 25-OH Vit D: 25-hydroxy vitamin D. *p*: Kruskal–Wallis test. Group 1: No dialysis history, carrying AKIN stage 3 criteria, Group 2: Ongoing dialysis treatment for any cause other than uremic encephalopathy, carrying AKIN stage 3 criteria, Group 3: Undergoing dialysis for uremic encephalopathy as per AKIN stage 3 criteria.

**Table 2:** Comparison of 24 h follow-up biochemical parameters of the groups according to AKIN stage 3.

	Group 1 (n = 23)		Group 2 (n = 17)		Group 3 (n = 9)		
Biochemical markers (serum)	Mean ± SD	Median (min–max)	Mean ± SD	Median (min–max)	Mean ± SD	Median (min–max)	*P*
**Creatinine (mg/dL)**	3.9 ± 1.6	3.6 (1.4–6.8)	6.5 ± 2.5	6.7 (3.4–10.7)	4.9 ± 1.4	4.5 (3.7–8.2)	**0.001**
**Urea (mg/dL)**	151.0 ± 70.4	132.0 (29.3–356.0)	196.4 ± 92.2	194.0 (91.8–405.0)	159.5 ± 41.6	161.0 (76.8–217.6)	0.354
**Albumin (g/dL)**	3.6 ± 0.6	3.6 (2.6–4.9)	3.3 ± 0.7	3.2 (2.2–4.7)	2.9 ± 0.6	2.7 (2.4–4.2)	**0.045**
**Calcium (mg/dL)**	8.9 ± 0.7	8.9 (7.1–10.5)	8.3 ± 0.8	8.4 (6.2–9.6)	7.2 ± 1.7	7.8 (3.5–8.7)	**0.002**
**Ionized calcium mmol/L)**	1.2 ± 0.1	1.2 (1.0–1.3)	1.1 ± 0.3	1.1 (0.3–1.9)	0.9 ± 0.2	1.1 (0.6–1.2)	**0.0001**

Mean: Average, SD: Standard deviation. *p*: Kruskal–Wallis test. Group 1: No dialysis history, carrying AKIN stage 3 criteria, Group 2: Ongoing dialysis treatment for any cause other than uremic encephalopathy, carrying AKIN stage 3 criteria, Group 3: Undergoing dialysis for uremic encephalopathy as per AKIN stage 3 criteria.

**Table 3:** Comparison of 72 h follow-up biochemical parameters of the groups according to AKIN stage 3.

	Group 1 (n = 23)		Group 2 (n = 17)		Group 3 (n = 9)		
Biochemical markers (serum)	Mean ± SD	Median (min–max)	Mean ± SD	Median (min–max)	Mean ± SD	Median (min–max)	*p*
**Creatinine (mg/dL)**	3.3 ± 1.6	3.2 (1.1–7.9)	5.3 ± 2.9	5.2 (1.3–11.3)	4.9 ± 0.9	4.9 (3.5–6.1)	**0.008**
**Urea (mg/dL)**	129.2 ± 42.9	125.0 (54.9–236.0)	146.8 ± 62.4	130.0 (51.8–279.0)	151.6 ± 57.7	174.0 (74.8–219.0)	0.626
**Albumin (g/dL)**	3.7 ± 0.8	3.7 (2.4–4.8)	3.3 ± 0.7	3.2 (2.2–4.7)	2.8 ± 0.4	2.6 (2.4–3.5)	**0.005**
**Calcium (mg/dL)**	8.9 ± 0.7	9.2 (7.1–9.9)	8.5 ± 1.1	8.5 (6.0–10.5)	7.1 ± 1.3	7.5 (4.7–8.5)	**0.001**
**Ionized calcium (mmol/L)**	1.2 ± 0.1	1.2 (1.0–1.4)	1.1 ± 0.1	1.1 (0.8–1.3)	0.9 ± 0.2	0.9 (0.7–1.2)	**0.0001**

Mean: Average, SD: Standard deviation. *p*: Kruskal–Wallis test. Group 1: No dialysis history, carrying AKIN stage 3 criteria, Group 2: Ongoing dialysis treatment for any cause other than uremic encephalopathy, carrying AKIN stage 3 criteria, Group 3: Undergoing dialysis for uremic encephalopathy as per AKIN stage 3 criteria.
